# The Effect of Stereochemistry on Sodium Ion Complexation in Nucleoside-Metallacarborane Conjugates

**DOI:** 10.1155/2010/196064

**Published:** 2010-07-01

**Authors:** Agnieszka B. Olejniczak, Jan Milecki, Grzegorz Schroeder

**Affiliations:** ^1^Laboratory of Molecular Virology and Biological Chemistry, Institute for Medical Biology of the Polish Academy of Sciences, Lodowa 106, 93-232 Łódź, Poland; ^2^Faculty of Chemistry, Adam Mickiewicz University, Grunwaldzka 6, 60-780 Poznań, Poland

## Abstract

Conjugates of purine and pyrimidine nucleosides: thymidine and 2′-deoxyguanosine with cobalt-metallacarborane were studied for their sodium ion complexing properties. Formation of stable complexes of 1 : 1 stoichiometry was proved by ESI MS spectroscopy and ^23^Na NMR. Equilibrium constants and energies of complex formation were calculated. Complexation of alkali-metals by nucleoside-metallacarborane conjugates may affect the physicochemical and biological properties of the conjugates and should be taken into consideration during biological evaluation of these types of modifications.

## 1. Introduction

The incorporation of metal centres into nucleosides and then nucleic acids provides an exceptional opportunity to create a new type of functionalised bioinorganic material that merges the information bearing property of nucleic acids with the electronic, magnetic, or optical properties of metals [1, 2]. A productive approach for the incorporation of metal centres into nucleic acids must be versatile, modular, and synthetically simple. Therefore, often metal complexes containing nucleosides are used as modified modules. On the other hand, metal containing nucleosides and their analogues have themselves great potential as chemotherapeutics and tools in molecular biology [3]. 

**Scheme 1:**
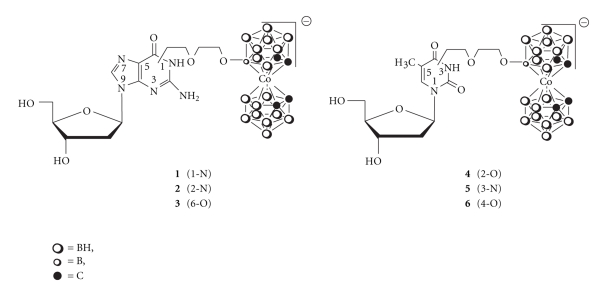
Schematic structures of the conjugates: ({5-[3-cobalt bis(1,2-dicarbollide)-8-yl]-3-oxa-pentoxy}-2′-O-deoxyguanosine)^−^, **1**–**3** and ({5-[3-cobalt bis(1,2-dicarbollide)-8-yl]-3-oxa-pentoxy}thymidine)^−^, **4**–**6.**

Recently, we described a general method for the synthesis of conjugates of four canonical nucleosides: T, dC, dA, dG, and [3-cobalt bis (1,2-dicarbollido(-1)] ion [4]. The incorporation of the thymidine conjugate into a DNA-oligomer *via* the phosphoramidite method has also been shown [5]. In the case of 2′-deoxyguanosine and thymidine, all possible regioisomers 1-N (**1**), 2-N (**2**), 6-O (**3**), 2-O (**4**), 3-N (**5**), and 4-O (**6**) of the conjugate: ({5-[3-cobalt bis (1,2-dicarbollide)-8-yl]-3-oxa-pentoxy}-2′-*O*-deoxyguanosine)^−^ and ({5-[3-cobalt bis(1,2-dicarbollide)-8-yl]-3-oxa-pentoxy}thymidine)^−^, respectively, have been obtained (Scheme 1).

The presence of several coordination centres in the conjugate, including those in the 3-oxa-pentoxy linker, introduces the possibility of metal complexation through noncovalent interactions. The ability to form such complexes may not only affect the *syn*/*anti* equilibrium of the nucleoside conjugate but may also change its entire conformation. In turn, the physicochemical and biological properties of the modified nucleoside may be affected, as well as the properties of the DNA-oligomers bearing the modification [6, 7]. The spacer between the metallacarborane and the nucleobase in **1–6** (Scheme 1) can be considered a short fragment of polyethylene glycol (PEG) [in this case it is di(ethylene glycol)]; this type of spacer is advantageous for its high degree of conformational freedom and biocompatibility. PEG is widely used as a covalent modifier of biological macromolecules and a tether for preparing bioconjugates of various biologically important molecules, including proteins, peptides, lipids, and DNA-oligonucleotides [8]. Often, the complexation properties of the PEG are neglected or assumed to be unimportant; the linker is usually considered flexible and lacking specific steric preferences. These assumptions are not always valid, however.

In this communication, we focus on the varying abilities of the conjugates **1–6** to complex a sodium ion and form organised spatial structures with the linker and nucleobase in a highly cooperative manner (Table 1). 

Electrospray ionisation mass spectrometry (ESI MS), ^23^Na NMR, and theoretical calculations are typically used to study the metal cation complexation properties of ionophores and host-guest systems [9–12]. For example, the equilibrium between two forms of complexes that arise from different possible folding states of a polyether chain has been postulated for uridine derivatives [12]. This equilibrium supports a more stable complex than systems in which no such exchange is possible. We used similar methodology in the present study. Recent work showing that PEG attached to a simple boron cluster systems can also specifically coordinate sodium ions [13–18] provides additional rational for the study of more complicated and biologically relevant nucleoside/metallacarborane conjugates.

## 2. Experimental

### 2.1. Materials

The formulas of the molecules that were studied are shown in Scheme 1. All compounds were synthesized according to the described procedure [4]. Sodium perchlorate was purchased from Aldrich Chemical Co. and was dried in vacuum. The solutions of the complexes with sodium ions were prepared by dissolving the respective compound and sodium perchlorate in anhydrous methanol at molar ratios of 1 : 5. The final concentration of compounds **1–6** was 1 × 10^−4^ mol/L. All of the preparations and transfers of solutions were carried out in a carefully dried glovebox under an atmosphere of nitrogen.

### 2.2. Mass Spectrometry—ESI MS

The Electrospray-Ionisation Mass Spectra (ESI MS) were recorded on a ZQ Waters/Micromass Mass Spectrometer (Manchester, UK) with quadruple analyser. The following parameters were used: source potential ESI on capillaries: 3 kV; voltage on focal plate: 0.5 V; voltage on extractor: 4 V; the cone voltage (cv): 30 V; ion fragmentation was examined with 30, 70, 90, and 130 V (cv); source temperature: 120°C; evaporation temperature: 300°C; nitrogen was used as a spraying, and drying gas with a flow rate of 80 and 300 Lh^−1^. 

ESI mass spectra of the negative ions of the studied compounds in methanol solutions were registered in MCA mode (Multi Channel Acquisition) with an *m*/*z* = 100–1000 interval. A typical spectrum obtained was the average of 10 scans with a 0.6 s interval. The solutions studied were introduced into the ionization source (at a flow rate 40 *μ*L min ^−1^) through a Harvard's pump.

### 2.3. NMR Spectroscopy—^23^ *N* *a* *N* *M* *R*

^23^Na NMR spectra were recorded using the following parameters: sfrq = 79.373 kHz; sw = 20000 Hz; pw = 70°; at = 1.0 s; *T* = 293.0 K and a 1 mol/L solution of NaCl/D_2_O as the external standard. Digital resolution = 0.7 Hz per point. No zero filling or window function was used.

### 2.4. PM5 Semiempirical Calculations

PM5 semiempirical calculations were performed using the standard Mopac 2002 Program, with full geometry optimization and without any symmetry constrains. The optimized geometries of the molecules were calculated setting the gradient in the hypersurface of energy to be lower (in module) than 0.05 kcal/mol, in order to assure good quality results [19].

## 3. Results and Discussion

Compounds **1–6** (Scheme 1) were obtained according to the original procedure described by us recently [4]. To study the effect of the location of the modification within the nucleobase on sodium ion complexation, the derivatives of thymidine and 2′-deoxyguanosine **1–6** were chosen due to ability to form regioisomeric alkylation products. In the case of thymidine and 2′-deoxyguanosine deprotonation at 3-N and 1-N, respectively, leads to ambident ion formation with the negative charge distributed over 2-O, 3-N, and 4-O for protected thymidine and 1-N and 6-O for protected 2′-deoxyguanosine which leads to the formation of the regioisomers. In the case of 2′-deoxycytidine and 2′-deoxyadenosine the only side susceptible to deprotonation is an exo-amine group which leads to one product only [4].

Alkali-metal cation complexation is a well known property of ethers [12]. It is also known that the 3-oxa-pentoxy linker present in** 1–6** favours the chelation of cations such as Na^+^ and produces five-member rings. Indeed, it was shown that in the sodium salt Na{3,3′-Co(8-(CH_2_CH_2_O)_2_ CH_2_CH_3_}-1,2-C_2_B_9_H_10_)(1′,2′-C_2_B_9_H_11_)] the chain contributes three oxygen atoms to the coordination of Na^+^ and that the metallacarborane cluster provides three extra B–H coordination sites [14, 17, 18].

Our study has shown that in conjugates **1–6**, the nucleobase of the nucleoside residue can contribute additional coordination sites to sodium ion complexation. While the participation of the carborane cluster in sodium ion complexation has been observed by others [17, 18], it can be neglected in this case, since the interactions between the cation and free electron pairs of the spacer oxygen atoms and the oxygen and/or nitrogen atoms of nucleotide base dominate over the weaker boron-hydrogen atom interactions. This is consistent with observation that although evidence for B–H^…^Na^+^ interactions in the solid state is given by X-ray analysis of the sodium salts, no proof of its existence in solution has been found in the ^1^H{^11^B} NMR spectrum at room temperature [17, 18]. 

**Figure 1:**
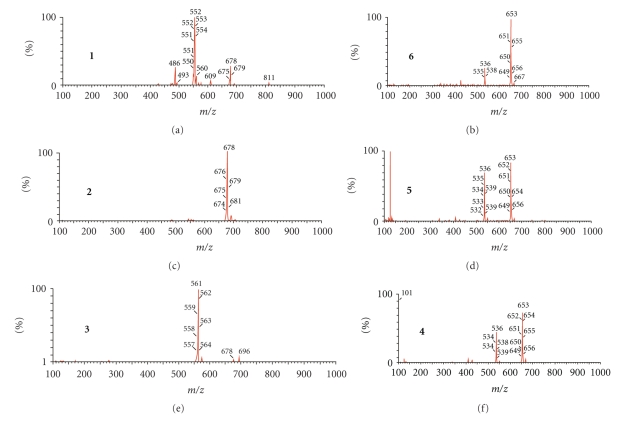
Example ESI MS spectra for compounds **1–3** (cv = 30 V) and **4–6** (cv = 70 V), in methanol, negative regions.

Mass spectrometry is an excellent analytical method for establishing the substitution site. The fragmentation patterns of the compounds are strongly influenced by the locations of the side groups. ESI MS spectra of methanol solutions show a fragmentation path of the studied compounds that depends strongly on the position of the substituent within the nucleoside conjugate (Figure 1). 

Due to the large number of boron atoms in the two isotopic forms (^10^B: 19.9% and ^11^B: 80.1%), the signals corresponding to the ions containing the boron cluster in the ESI MS spectra are quite complicated albeit on the other hand, this fact enables easy identification of the signals and presents an excellent analytical tool. For analytical purposes, the spectra that were run at cv = 30 V can be applied to compounds **1–3** (Figure 1, Table 2), where distinct differences in the spectra was observed.

**Figure 2:**
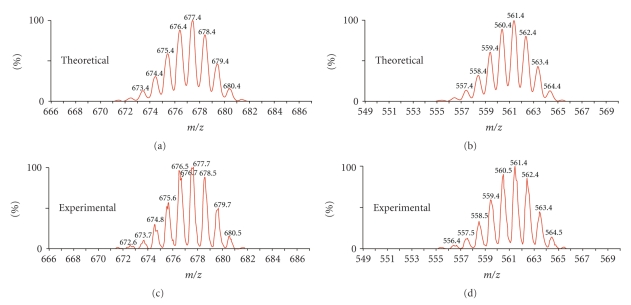
The theoretical and experimental *m*/*z* = 678 ESI MS signals (left) and *m*/*z* = 561 ESI MS signals (right) for compound **3**, negative regions.

Compounds **4–6** can be identified in the spectra run at cv = 70 V by diverse fragmentation and changes in the intensities of the signals (Figure 1, Table 2). The calculated and experimental fragmentation spectra for compound **3** are shown as an example in Figure 2.

The structure of the fragment ions A-E (Table 2) was not studied in detail. However, they can be grouped into two classes: ions containing boron, derived from the mother metallacarborane: 3-cobalt bis (1, 2-dicarbollide) (A, C) (Table 2) and ions corresponding to the nucleoside unit of the conjugate or its fragments (B, D, E) (Table 2).

**Figure 3:**
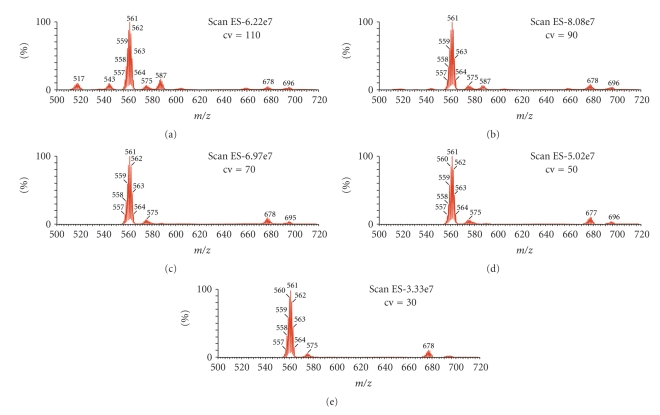
ESI MS spectra for compound **3** *m*/*z* = 500–700 at various cone voltages cv = 30, 50, 70, 90, 110 V, negative regions.

**Figure 4:**
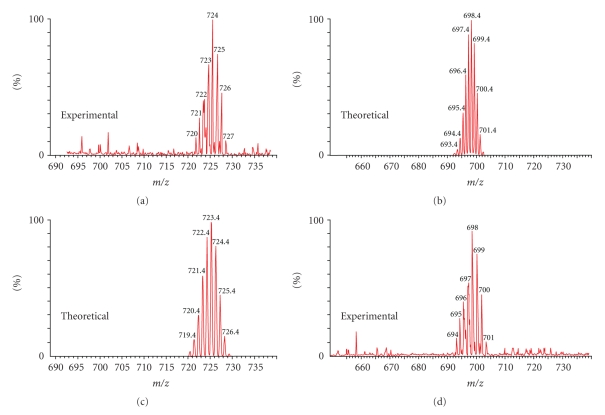
The experimental and theoretical *m*/*z* = 725 (B_18_C_18_CoH_41_N_5_O_6_Na_2_) ESI MS spectra of the complex of compound **3** (left), and *m*/*z* = 698 (B_18_C_18_CoH_42_N_2_O_7_Na_2_) ESI MS spectra of the complex of compound **4** (right), with NaClO_4_, positive regions.

The influence of the cv parameter on fragmentation for compound **3** is illustrated in Figure 3. It is clear that the system is highly stabilised, and therefore additional energy is not reflected in enhanced fragmentation. The addition of sodium ions drastically changes the spectra of the studied compounds. Signals corresponding to the disodio-derivatised molecules appear in the positive ions area (Figure 4). 

This observation stresses the necessity of careful consideration of ion complexation and its effect on the physicochemical and biological properties of the nucleoside-metallacarborane conjugates such as **1–6** and DNA-oligomers containing this type of modification. In addition, the discovered relationship between the regiochemistry of the nucleoside-metallacarborane conjugates and their ESI-MS characteristics allows for a rapid and convenient analysis and identification of regioisomers *via *simple mass spectrometry analysis.

^23^Na NMR spectra were measured for all the systems studied. The chemical shift differences that were found for free NaClO_4_ and its 1 : 1 complex with the compounds **1–6** ranged from 0.27 to 0.35 ppm in acetonitrile. The ^23^Na NMR titration method gave complex stability constants of l gK = 0.82–1.25. Theoretical calculations [19] using the PM5 method for the complexing core of the studied compounds with sodium ions (Table 1) confirm that they can form stable ionic omplexes. 

Molecular Orbital Package (MOPAC) provides a choice of methods for computing electronic properties of molecules. In this paper we carried out PM5 calculations using the MOPAC 2002 program included in the CACHE suite. The optimized geometries of molecules were calculated setting the gradient in the hypersurface of energy to be lower (in module) than 0.05 kcal/mol, in order to assure good quality results. The results of computational method calculations, PM5 (parametric method 5) give only models, and there is no advantage in rigorously solving Schrödinger's equation for a large system if that system has had to be abbreviated in order to make the calculations tractable. Semiempirical methods are thus seen to be well balanced: they are accurate enough to have useful predictive powers, yet are sufficiently fast to allow large systems to be studied. PM5 method self-consistent field (SCF) method, takes into account electrostatic repulsion and exchange stabilization, and, in them, all calculated integrals are evaluated by approximate means. Further, they use a restricted basis set of one s and three px, py, and pz orbitals per atom and ignore overlap integrals in the secular equation. PM5 Hamiltonian is the method for computing electronic properties of molecules, it is more accurate and include all main group elements, such as B and Na, not previously available in PM3.

Theoretical calculations [19] using PM5 method for the complexing core of the conjugates **1–6** where *X * = metallacarborane (Table 1) was performed. Formation of the stable ionic complex was confirmed. Depending on the site at which the nucleobase of the nucleoside conjugate is substituted with the metallacarborane, different enthalpy of complex formation was observed. The role of heteroatom present in the nucleobase is crucial for formation of stable complex with sodium ion. The energy gain from the complexation of sodium ions was established at 254–335 kJ/mol for complexes of conjugates **1–6** (*X*  =  3-cobalt bis(carbollide), Table 1) and at 234–305 kJ/mol for the complexing core of the conjugates **1–6** using simplified model contain methyl group at the terminal oxygen atom (*X* = CH_3_). Though enthalpies of complex formation (Δ*H*_*f*_) [kJ/mol] are similar in both cases, difference between *H*_*f*_ [kJ/mol] for complexes with sodium ion and uncomplexed sodium ion are noteworthy: −1634.85 to −1338.94 and −1299.73 to −1041.43 kJ/mol for *X* = 3-cobalt bis(carbollide) and −306.8 to 80.04 and −38.43 to 363.61 kJ/mol, for *X* = CH_3_. The structures that were assigned by the PM5 method (Table 1) are consistent with ESI MS and ^23^Na NMR analysis.

## 4. Conclusion

A new type of nucleoside-metallacarborane complex with sodium ions was observed. The di(ethyleneglycol) chain that is applied as a linker with the borane-cobalt marker plays a fundamental role in the formation of these complexes. Regiochemistry and the location of the linker within the nucleobase of nucleoside unit play crucial roles in the formation of the intramolecular complex with the sodium ion. This observed phenomenon may affect the properties of biomolecules, such as nucleosides or porphyrines [20, 21] that are modified with a metallacarborane group linked *via* a di(ethyleneglycol) (polyoxyethylene) linker, as well as the recently described anti-HIV metallacarborane derivatives [22], and should be taken into consideration when evaluating biological properties of these types of conjugates.

## Figures and Tables

**Table 1:** Calculated enthalpy of formation (Δ*H*_*f*_) [kJ/mol] for complexing core of complexes of **1–6** with sodium cations (PM5 method), where *X* = 3-cobalt bis(1, 2-dicarbollide).

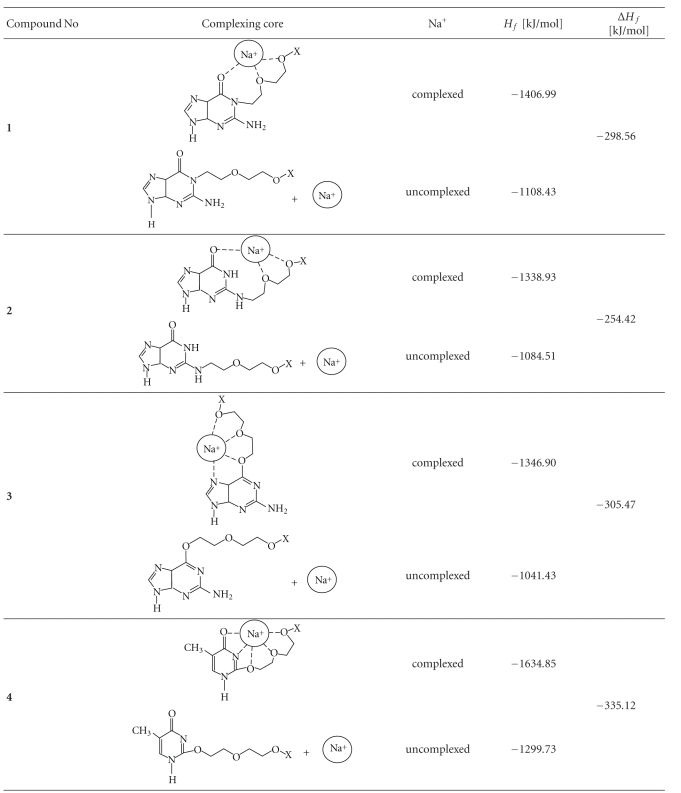 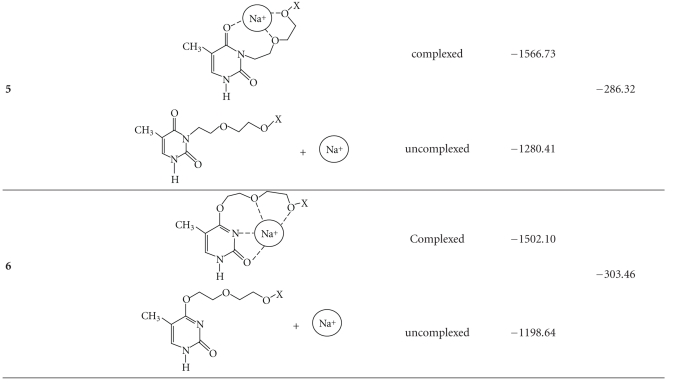

**Table 2:** The main peaks in the ESI mass spectra recorded for negative ions and relative intensity of signals for metallacarborane-2′-deoxyguanosine conjugates **1–3**, and metallacarborane-thymidine conjugates **4–6**, at various cone voltages.

		*m/z*			
No	Molecular formula	Cone voltage (cv) [V]			
	MW*	30	50	70	90
**1**	C_18_H_41_N_5_O_6_B_18_Co MW 677	486 (22%); (M-C)^−^ 552 (100%); (M-A)^−^ 678 (22%); M^−^	552 (100%); (M-A)^−^ 678 (18%); M^−^	486 (20%); (M-C)^−^ 552 (20%); (M-A)^−^ 678 (100%); M^−^	486 (100%); (M-C)^−^ 552 (20%); (M-A)^−^ 678 (22%); M^−^

**2**	C_18_H_41_N_5_O_6_B_18_Co MW 677	678; M^−^	561 (20%); (M-B)^−^ 678 (100%); M^−^	561 (40%); (M-B)^−^ 678 (100%); M^−^	561 (80%); (M-B)^−^ 678 (100%); M^−^

**3**	C_18_H_41_N_5_O_6_B_18_Co MW 677	561 (100%); (M-B)^−^ 678 (trace); M^−^	561; (M-B)^−^ 677 (trace); M^−^	561; (M-B)^−^ 678 (trace); M^−^	561; (M-B)^−^ 678 (trace); M^−^

**4**	C_18_H_42_N_2_O_7_B_18_Co, MW 652	653; M^−^	536 (100%); (M-E)^−^ 653 (25%); M^−^	410 (15%); (M-D)^−^ 536 (80%); (M-E)^−^ 653 (100%); M^−^	410 (30%); (M-D)^−^ 536 (100%); (M-E)^−^ 653 (25%); M^−^

**5**	C_18_H_42_N_2_O_7_B_18_Co MW 652	653; M^−^	536 (10%); (M-E)^−^ 653 (100%); M^−^	410 (10%), (M-D)^−^ 536 (60%); (M-E)^−^ 653 (100%); M^−^	536 (90%); (M-E)^−^ 653 (100%); M^−^

**6**	C_18_H_42_N_2_O_7_B_18_Co MW 652	653; M^−^	410 (10%); (M-D)^−^ 536 (100%); (M-E)^−^ 653 (20%); M^−^	410 (10%); (M-D)^−^ 536 (20%); (M-E)^−^ 653 (100%); M^−^	410 (10%); (M-D)^−^ 536 (100%); (M-E)^−^ 653 (20%); M^−^

*Calculation of the theoretical molecular mass for compounds **1–6** was performed using option “Analyze Structure” in ChemDraw Program. The calculated masses provided in the manuscript correspond to molecular weight (MW) based on the average mass of the elements consisting natural isotopes.

Where (for **1–3**): M^−^ *m*/*z* = 678 (B_18_C_18_CoH_41_N_5_O_6_), (M-A)^−^ *m*/*z* = 552 (B_17_C_13_CoH_33_N_5_O_3_), (M-B)^−^ *m*/*z* = 561 (B_18_C_13_CoH_33_N_5_O_3_), (M-C)^−^ *m*/*z* = 486 (B_9_C_16_H_30_N_5_O_6_).

Where (for **4–6**): M^−^ *m*/*z* = 653 (B_18_C_18_CoH_42_N_2_O_7_), (M-D)^−^ *m*/*z* = 410 (B_18_C_8_CoH_29_O_2_), (M-E)^−^ *m*/*z* = 536 (B_18_C_13_CoH_34_N_2_O_4_).
